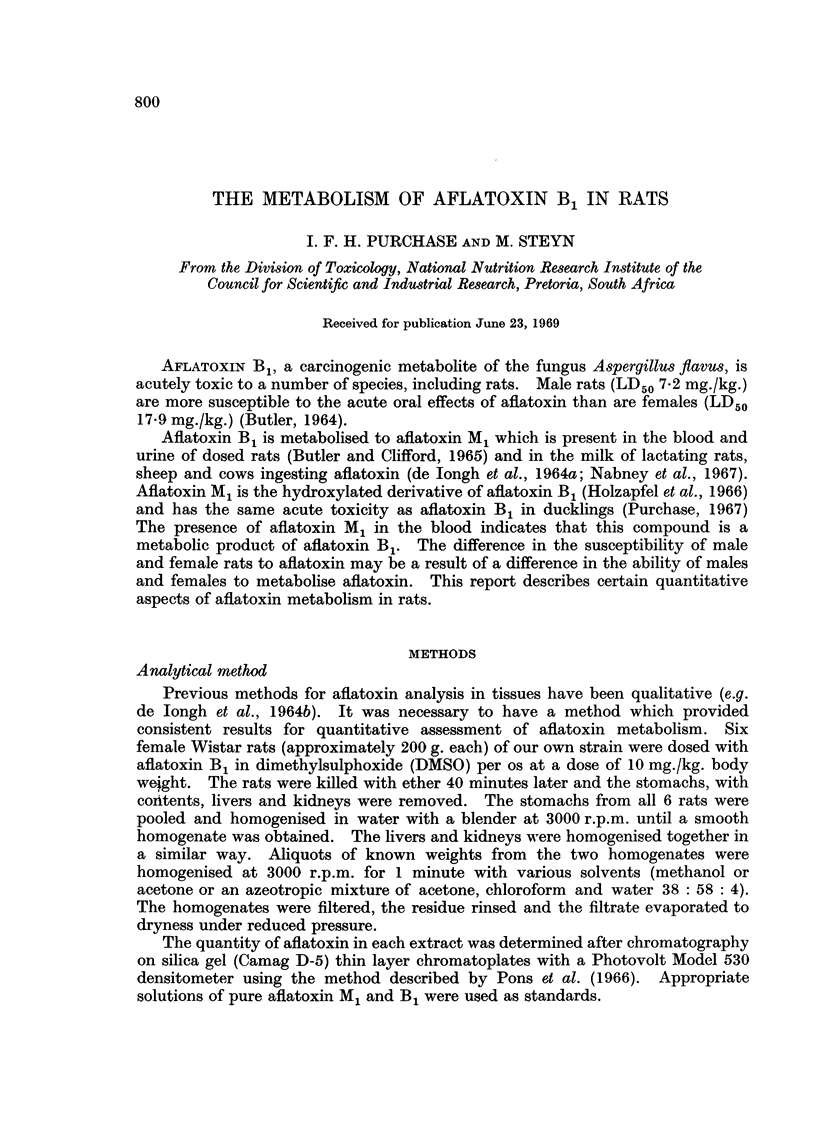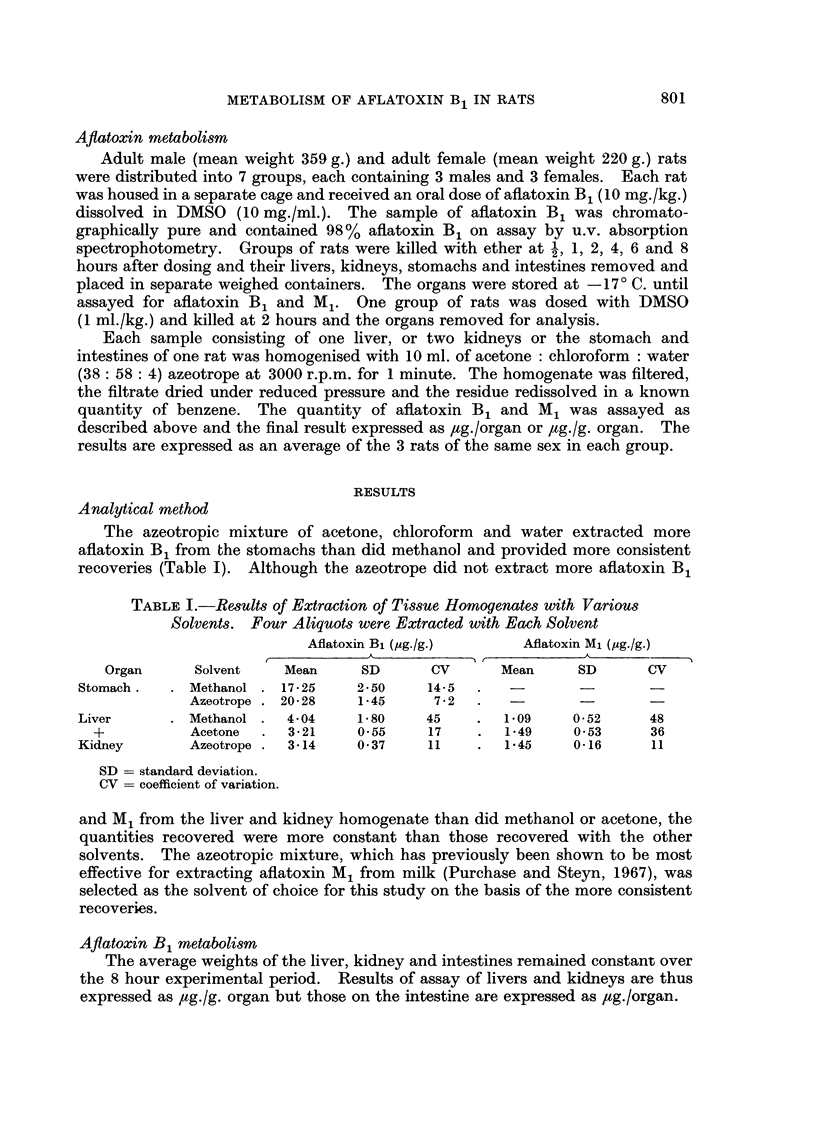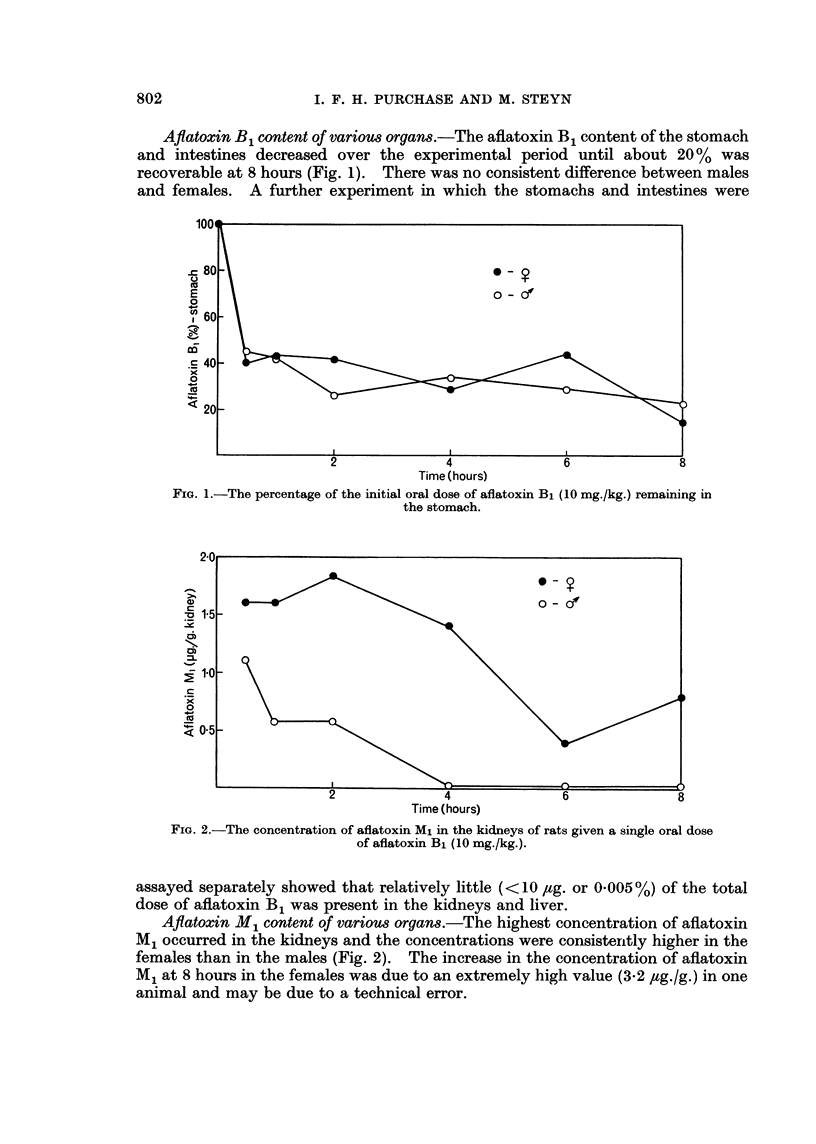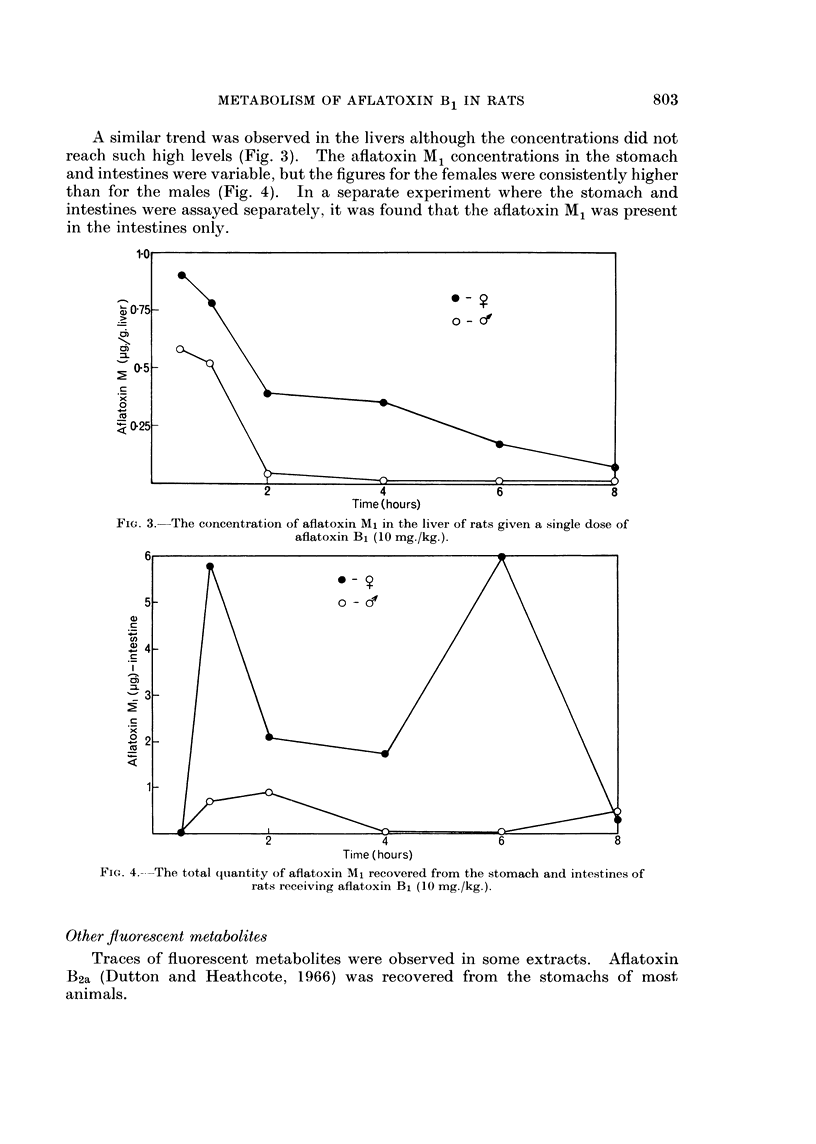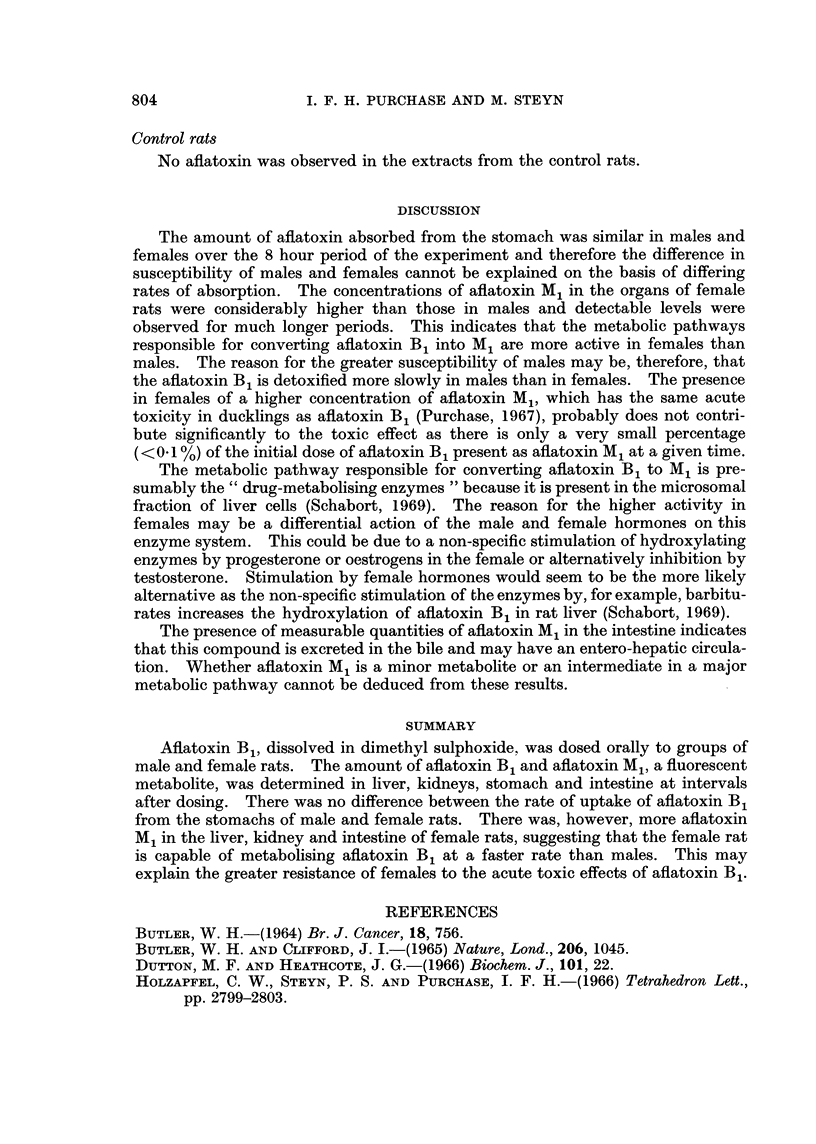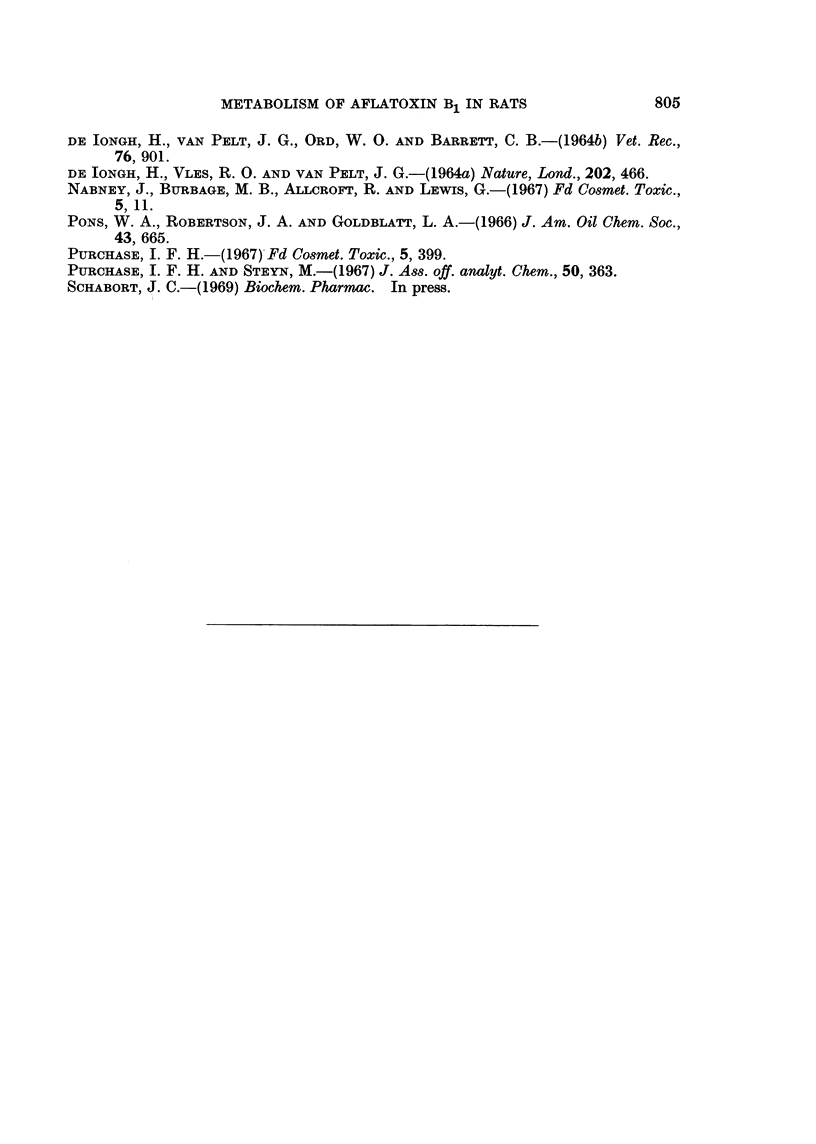# The metabolism of aflatoxin B1 in rats.

**DOI:** 10.1038/bjc.1969.99

**Published:** 1969-12

**Authors:** I. F. Purchase, M. Steyn


### THE METABOLISM OF AFLATOXIN $B_1$ IN RATS

I. F. H. PURCHASE AND M. STEYN

*From the Division of Toxicology, National Nutrition Research Institute of the Council for Scientific and Industrial Research, Pretoria, South Africa*

Received for publication June 23, 1969

AFLATOXIN $B_1$, a carcinogenic metabolite of the fungus *Aspergillus flavus*, is acutely toxic to a number of species, including rats. Male rats ($LD_{50}$ 7·2 mg./kg.) are more susceptible to the acute oral effects of aflatoxin than are females ($LD_{50}$ 17·9 mg./kg.) (Butler, 1964).

Aflatoxin $B_1$ is metabolised to aflatoxin $M_1$ which is present in the blood and urine of dosed rats (Butler and Clifford, 1965) and in the milk of lactating rats, sheep and cows ingesting aflatoxin (de Iongh *et al.*, 1964*a*; Nabney *et al.*, 1967). Aflatoxin $M_1$ is the hydroxylated derivative of aflatoxin $B_1$ (Holzapfel *et al.*, 1966) and has the same acute toxicity as aflatoxin $B_1$ in ducklings (Purchase, 1967) The presence of aflatoxin $M_1$ in the blood indicates that this compound is a metabolic product of aflatoxin $B_1$. The difference in the susceptibility of male and female rats to aflatoxin may be a result of a difference in the ability of males and females to metabolise aflatoxin. This report describes certain quantitative aspects of aflatoxin metabolism in rats.

### METHODS

### *Analytical method*

Previous methods for aflatoxin analysis in tissues have been qualitative (*e.g.* de Iongh *et al.*, 1964*b*). It was necessary to have a method which provided consistent results for quantitative assessment of aflatoxin metabolism. Six female Wistar rats (approximately 200 g. each) of our own strain were dosed with aflatoxin $B_1$ in dimethylsulphoxide (DMSO) per os at a dose of 10 mg./kg. body weight. The rats were killed with ether 40 minutes later and the stomachs, with contents, livers and kidneys were removed. The stomachs from all 6 rats were pooled and homogenised in water with a blender at 3000 r.p.m. until a smooth homogenate was obtained. The livers and kidneys were homogenised together in a similar way. Aliquots of known weights from the two homogenates were homogenised at 3000 r.p.m. for 1 minute with various solvents (methanol or acetone or an azeotropic mixture of acetone, chloroform and water 38 : 58 : 4). The homogenates were filtered, the residue rinsed and the filtrate evaporated to dryness under reduced pressure.

The quantity of aflatoxin in each extract was determined after chromatography on silica gel (Camag D-5) thin layer chromatoplates with a Photovolt Model 530 densitometer using the method described by Pons *et al.* (1966). Appropriate solutions of pure aflatoxin $M_1$ and $B_1$ were used as standards.

*Aflatoxin metabolism*

Adult male (mean weight 359 g.) and adult female (mean weight 220 g.) rats were distributed into 7 groups, each containing 3 males and 3 females. Each rat was housed in a separate cage and received an oral dose of aflatoxin $B_1$ (10 mg./kg.) dissolved in DMSO (10 mg./ml.). The sample of aflatoxin $B_1$ was chromatographically pure and contained 98% aflatoxin $B_1$ on assay by u.v. absorption spectrophotometry. Groups of rats were killed with ether at $\frac{1}{2}$, 1, 2, 4, 6 and 8 hours after dosing and their livers, kidneys, stomachs and intestines removed and placed in separate weighed containers. The organs were stored at $-17°$ C. until assayed for aflatoxin $B_1$ and $M_1$. One group of rats was dosed with DMSO (1 ml./kg.) and killed at 2 hours and the organs removed for analysis.

Each sample consisting of one liver, or two kidneys or the stomach and intestines of one rat was homogenised with 10 ml. of acetone : chloroform : water (38 : 58 : 4) azeotrope at 3000 r.p.m. for 1 minute. The homogenate was filtered, the filtrate dried under reduced pressure and the residue redissolved in a known quantity of benzene. The quantity of aflatoxin $B_1$ and $M_1$ was assayed as described above and the final result expressed as $\mu$g./organ or $\mu$g./g. organ. The results are expressed as an average of the 3 rats of the same sex in each group.

### RESULTS

*Analytical method*

The azeotropic mixture of acetone, chloroform and water extracted more aflatoxin $B_1$ from the stomachs than did methanol and provided more consistent recoveries (Table I). Although the azeotrope did not extract more aflatoxin $B_1$

TABLE I.—*Results of Extraction of Tissue Homogenates with Various Solvents. Four Aliquots were Extracted with Each Solvent*

| | | Aflatoxin $B_1$ ($\mu$g./g.) | | | Aflatoxin $M_1$ ($\mu$g./g.) | | |
|---|---|---|---|---|---|---|---|
| Organ | Solvent | Mean | SD | CV | Mean | SD | CV |
| Stomach | Methanol | 17·25 | 2·50 | 14·5 | — | — | — |
| | Azeotrope | 20·28 | 1·45 | 7·2 | — | — | — |
| Liver | Methanol | 4·04 | 1·80 | 45 | 1·09 | 0·52 | 48 |
| + | Acetone | 3·21 | 0·55 | 17 | 1·49 | 0·53 | 36 |
| Kidney | Azeotrope | 3·14 | 0·37 | 11 | 1·45 | 0·16 | 11 |

SD = standard deviation.
CV = coefficient of variation.

and $M_1$ from the liver and kidney homogenate than did methanol or acetone, the quantities recovered were more constant than those recovered with the other solvents. The azeotropic mixture, which has previously been shown to be most effective for extracting aflatoxin $M_1$ from milk (Purchase and Steyn, 1967), was selected as the solvent of choice for this study on the basis of the more consistent recoveries.

*Aflatoxin $B_1$ metabolism*

The average weights of the liver, kidney and intestines remained constant over the 8 hour experimental period. Results of assay of livers and kidneys are thus expressed as $\mu$g./g. organ but those on the intestine are expressed as $\mu$g./organ.

*Aflatoxin $B_1$ content of various organs.*—The aflatoxin $B_1$ content of the stomach and intestines decreased over the experimental period until about 20% was recoverable at 8 hours (Fig. 1). There was no consistent difference between males and females. A further experiment in which the stomachs and intestines were

FIG. 1.—The percentage of the initial oral dose of aflatoxin $B_1$ (10 mg./kg.) remaining in the stomach.

FIG. 2.—The concentration of aflatoxin $M_1$ in the kidneys of rats given a single oral dose of aflatoxin $B_1$ (10 mg./kg.).

assayed separately showed that relatively little ($<10$ $\mu$g. or 0·005%) of the total dose of aflatoxin $B_1$ was present in the kidneys and liver.

*Aflatoxin $M_1$ content of various organs.*—The highest concentration of aflatoxin $M_1$ occurred in the kidneys and the concentrations were consistently higher in the females than in the males (Fig. 2). The increase in the concentration of aflatoxin $M_1$ at 8 hours in the females was due to an extremely high value (3·2 $\mu$g./g.) in one animal and may be due to a technical error.

A similar trend was observed in the livers although the concentrations did not reach such high levels (Fig. 3). The aflatoxin $M_1$ concentrations in the stomach and intestines were variable, but the figures for the females were consistently higher than for the males (Fig. 4). In a separate experiment where the stomach and intestines were assayed separately, it was found that the aflatoxin $M_1$ was present in the intestines only.

Fig. 3.—The concentration of aflatoxin $M_1$ in the liver of rats given a single dose of aflatoxin $B_1$ (10 mg./kg.).

Fig. 4.—The total quantity of aflatoxin $M_1$ recovered from the stomach and intestines of rats receiving aflatoxin $B_1$ (10 mg./kg.).

*Other fluorescent metabolites*

Traces of fluorescent metabolites were observed in some extracts. Aflatoxin $B_{2a}$ (Dutton and Heathcote, 1966) was recovered from the stomachs of most animals.

*Control rats*

No aflatoxin was observed in the extracts from the control rats.

### DISCUSSION

The amount of aflatoxin absorbed from the stomach was similar in males and females over the 8 hour period of the experiment and therefore the difference in susceptibility of males and females cannot be explained on the basis of differing rates of absorption. The concentrations of aflatoxin $M_1$ in the organs of female rats were considerably higher than those in males and detectable levels were observed for much longer periods. This indicates that the metabolic pathways responsible for converting aflatoxin $B_1$ into $M_1$ are more active in females than males. The reason for the greater susceptibility of males may be, therefore, that the aflatoxin $B_1$ is detoxified more slowly in males than in females. The presence in females of a higher concentration of aflatoxin $M_1$, which has the same acute toxicity in ducklings as aflatoxin $B_1$ (Purchase, 1967), probably does not contribute significantly to the toxic effect as there is only a very small percentage ($<0{\cdot}1\%$) of the initial dose of aflatoxin $B_1$ present as aflatoxin $M_1$ at a given time.

The metabolic pathway responsible for converting aflatoxin $B_1$ to $M_1$ is presumably the " drug-metabolising enzymes " because it is present in the microsomal fraction of liver cells (Schabort, 1969). The reason for the higher activity in females may be a differential action of the male and female hormones on this enzyme system. This could be due to a non-specific stimulation of hydroxylating enzymes by progesterone or oestrogens in the female or alternatively inhibition by testosterone. Stimulation by female hormones would seem to be the more likely alternative as the non-specific stimulation of the enzymes by, for example, barbiturates increases the hydroxylation of aflatoxin $B_1$ in rat liver (Schabort, 1969).

The presence of measurable quantities of aflatoxin $M_1$ in the intestine indicates that this compound is excreted in the bile and may have an entero-hepatic circulation. Whether aflatoxin $M_1$ is a minor metabolite or an intermediate in a major metabolic pathway cannot be deduced from these results.

### SUMMARY

Aflatoxin $B_1$, dissolved in dimethyl sulphoxide, was dosed orally to groups of male and female rats. The amount of aflatoxin $B_1$ and aflatoxin $M_1$, a fluorescent metabolite, was determined in liver, kidneys, stomach and intestine at intervals after dosing. There was no difference between the rate of uptake of aflatoxin $B_1$ from the stomachs of male and female rats. There was, however, more aflatoxin $M_1$ in the liver, kidney and intestine of female rats, suggesting that the female rat is capable of metabolising aflatoxin $B_1$ at a faster rate than males. This may explain the greater resistance of females to the acute toxic effects of aflatoxin $B_1$.

### REFERENCES

BUTLER, W. H.—(1964) *Br. J. Cancer*, **18**, 756.
BUTLER, W. H. AND CLIFFORD, J. I.—(1965) *Nature, Lond.*, **206**, 1045.
DUTTON, M. F. AND HEATHCOTE, J. G.—(1966) *Biochem. J.*, **101**, 22.
HOLZAPFEL, C. W., STEYN, P. S. AND PURCHASE, I. F. H.—(1966) *Tetrahedron Lett.*, pp. 2799–2803.

de Iongh, H., van Pelt, J. G., Ord, W. O. and Barrett, C. B.—(1964*b*) *Vet. Rec.*, **76**, 901.
de Iongh, H., Vles, R. O. and van Pelt, J. G.—(1964*a*) *Nature, Lond.*, **202**, 466.
Nabney, J., Burbage, M. B., Allcroft, R. and Lewis, G.—(1967) *Fd Cosmet. Toxic.*, **5**, 11.
Pons, W. A., Robertson, J. A. and Goldblatt, L. A.—(1966) *J. Am. Oil Chem. Soc.*, **43**, 665.
Purchase, I. F. H.—(1967) *Fd Cosmet. Toxic.*, **5**, 399.
Purchase, I. F. H. and Steyn, M.—(1967) *J. Ass. off. analyt. Chem.*, **50**, 363.
Schabort, J. C.—(1969) *Biochem. Pharmac.* In press.